# Supplementary material for: Differential expression of spatiotemporal sleep spindle clusters in aging
Source: Sleep Adv. 2025 Nov 25;6(4):zpaf084. doi: 10.1093/sleepadvances/zpaf084 (PMC12721383; doi:10.1093/sleepadvances/zpaf084)
Supplement: Supplementary_materials_zpaf084 [file supplementary_materials_zpaf084.pdf]

Differential expression of spatiotemporal sleep spindle clusters in ageing

SUPPLEMENTARY MATERIALS

Liisa Raud[1], Martijn Smits[1,2], Markus H. Sneve[1], Hedda T. Ness[1], Line Folvik[1], Björn Rasch[3], Anders M. Fjell[1,4]

1 Center for Lifespan Changes in Brain and Cognition, Department of Psychology, University of Oslo, 0373 Oslo, Norway

2 Clinical Neuroscience Laboratory, Department of Psychology, Norwegian University of Science and Technology, 7491 Trondheim, Norway

3 Department of Psychology, Division of Cognitive Biopsychology and Methods, University of Fribourg, Switzerland

4 Computational Radiology and Artificial Intelligence, Department of Radiology and Nuclear Medicine, Oslo University Hospital, 0372 Oslo, Norway

Corresponding author: Liisa Raud (liisa.raud@psykologi.uio.no)

Supplementary materials 1 (Sup1): Memory evaluation procedures

Source memory experiment. The source memory experiment contributed with three conceptually different scores, named “source recall”, “delayed source recall”, and “sleep gain”. For the source memory experiment, during the encoding phase, participants were shown pictures of 128 items, each together with a specific face or place (4 faces and 4 places in total). They were instructed to imagine an association between the item and the face/place, and then to evaluate the vividness of this association on the scale 1-4. Directly after the encoding task, their learning performance was tested with a forced choice task (AFC), in which the participant had to indicate, for each item, which face or place it had been paired with. After 12 hours, participants returned for a retrieval test, in which they were shown each item and several new items, and they had to indicate whether it had been previously associated with a face, place, they did not remember, or the item was new. The experimental memory score (“source recall”) was calculated by counting the correctly remembered face/place associations, subtracting the number of incorrect associations, divided by the total number of items, and lastly, averaged over the wake and sleep sessions for more robust estimation. This score thus reflects the percentage of correctly remembered associations after 12 hours, corrected for guessing. Given that the retrieval task prompts for the context in which each item association was formed, it is putatively reflecting episodic source memory[1].

The second memory score (“delayed source recall”) was also derived from the experimental procedure, particularly the 8-alternative forced choice tasks (AFC-1-2-3). In this task, participants were shown each item together with all four faces and four places. They had to choose, which face or place the item had been associated with during the encoding phase. This test was administered three times: first directly after encoding, then after the 12-hour period (directly after the in-scanner retrieval task described previously), and lastly, after about six days using an online version of the task. The memory score was calculated as the percentage of

accurate associations in all three timepoints (hit-hit-hit), thus indicating the percentage of associations correctly remembered after six days, putatively reflecting the ability to form durable memories[2,3].

The third memory score, "sleep gain," represents the benefit of a 12-hour sleep period compared to the same duration of wakefulness and is calculated as the difference in source recall accuracy (corrected for guessing) between sleep and wake conditions. A positive value indicates improved memory performance after sleep relative to the awake period, serving as an index of sleep-driven memory consolidation. This retrieval test focused on category associations rather than specific faces and places, making it well-suited for assessing gist memory formation and consolidation, typically linked to sleep[4–6]. However, since this test was not administered immediately after learning, the sleep-wake difference might be influenced by baseline variations before the 12-hour period. To address this, we additionally calculated the sleep gain effect using AFC-1 (baseline) and AFC-2 test performance. First, AFC-2 performance was calculated as the percentage of items remembered at both time points, relative to baseline AFC-1 performance. Then, difference scores between sleep and wake conditions were calculated.

Ecological memory task. This task was a custom ecological memory test, designed to objectively measure everyday attentional and memory performance, and contributed with two memory scores: “everyday recall”, and “delayed everyday recall”. This was implemented in the separate lab-visit, when the participants came in for neuropsychological testing (not used in this study). During this session, the experimenters highlighted ten previously scripted specific behaviors or details in the environment, for example asking the participant to hold a pen during a procedure, pointing out the location of the restrooms, commenting on the type of the music played during waiting, etc. At the end of the session, the participants were asked to recall what they did from the beginning of session until the start of the session, including as much detail as possible. After this spontaneous narration, participants were probed directly about specific episodes that they

had omitted from their narratives. This procedure was repeated four weeks later via a phone call. With participants' consent, their narratives were recorded and later transcribed by the experimenter following a 13-item questionnaire. The items were coded 'correct' if remembered correctly, 'incorrect' if participant indicated they had forgotten or gave a wrong answer, or 'irrelevant' if the answer was indefinite or too abstract for direct transcription, or when there was ambiguity whether this action was correctly performed during the session. All irrelevant items were discarded, and the percentage of correct responses to the remaining items served as the fourth ("everyday recall") and fifth ("dealyed everyday recall") memory score, reflecting immediate and delayed recall, respectively.

Everyday Memory Questionnaire. The last score ("emq") was derived from the Everyday Memory Questionnaire[7,8], which measures self-reported memory failures in everyday life. This 28-item questionnaire probes participants for the frequency of daily memory lapses, such as loosing items, forgetting appointments, etc. We calculated the sum of all items, and higher sum scores indicate worse perceived memory problems.

Supplementary materials 2 (Sup2): Pruning of the clustering feature matrix

(1) Correlation-based pruning

Pearson correlation matrix was calculated across all continuous features and, of the pairs with r > 0.70, only one of the features were retained. As a result, five features were discarded: amplitude and root mean square (triangular correlations with absolute power), number of oscillations (correlated with duration), Petrosian fractal dimension (correlated with permutation entropy), and post-spindle spectral density intercept (correlated with the exponent).

(2) Post-hoc pruning

Second pruning of the feature matrix was done after initial results were obtained. First, it was noted that the categorical feature 'cycle' and continuous variable 'time relative to sleep onset' were highly discriminative between clusters. The clustering was thus repeated after omitting 'cycle' and retaining the continuous timing variable, to obtain results without the partial redundancy between these two features. Lastly, it appeared that clustering results were biased by the stage information, likely due to the inclusion of numerous inter-related post-spindle period variables. Therefore, the clustering was repeated after discarding all post-spindle period variables. We chose to report this analysis as the main results for several reasons: (1) the stage information was captured well also in the results without post-spindle variables, and (2) as the variables originated from the signal *after* the spindle events, there was ambiguity whether we were clustering spindles or merely differences in sleep stages.

Supplementary materials 3 (Sup3): Extraction of post-spindle signal variables

Welch's method was used to calculate the power spectrum of the post-spindle period and relative power with respect to the total power was extracted for the canonic frequency bands in human sleep: delta (0.5-4 Hz), theta (4-8 Hz), alpha (8-12 Hz), and sigma (12-16 Hz). Next, the offset and the exponent of the frequency spectra were estimated using Python *fooof* package[9] (v. 1.0.0). Lastly, the post-spindle signal complexity was captured by permutation entropy, Petrosian fractal dimension, and Katz fractal dimension, calculated with the Python *antropy* package[10] (v. 0.1.6). These measures indicate the non-linear complexity of the signal, and can roughly be interpreted to reflect the predictability and regularity of the signal[11], with higher complexity putatively indicating increased information processing[12,13]. Permutation entropy was chosen due its computational efficiency and Petrosian fractal dimension as it was ranking relatively high among the features for automatic sleep stage classification[14]. Katz fractal dimension was calculated, as it is considered more appropriate for detecting within participant state changes[15,16]

Supplementary materials 4 (Sup4): Memory performance differences between young and old age groups

Memory score	Explanation	old mean (sd)	Young mean (sd)	t (df)	Cohen's d	p-value
source recall	Experimental retrieval performance (%) after 12 hours	51 (24)	68 (20)	-3.45 (64)	-0.81	0.001*
delayed source recall	Experimental retrieval performance (%) after ~6 days	31 (16)	50 (23)	-4.30 (74)	-0.95	<0.001*
sleep gain	Difference between source recall (5) between sleep and awake conditions	5 (22)	6 (18)	-0.35 (62)	-0.080	0.730
ecological recall	Ecological memory test performance (%) at the end of the session	62 (19)	72 (15)	-2.37 (61)	-0.56	0.021*
dealyed ecological recall	Ecological memory test performance (%) four weeks later	73 (18)	80 (15)	-2.00 (62)	-0.470	0.049*
emq	Everyday memory questionnaire	54 (14)	66 (21)	-2.94 (72)	-0.640	0.004*

Table S1. All memory score comparisons. Memory performance and statistical comparisons between young (n = 43) and old (n = 34 age groups. The values for young and old are mean values, with standard deviations in the brackets. *indicates significant p-value at the alpha level of 0.05.

Supplementary materials 5 (Sup5): Source Memory Performance Separately for Sleep and Awake Conditions

Memory measure	Level of specificity	Young		Old		r sleep-awake
		**Sleep**	**Awake**	**Sleep**	**Awake**	
Baseline	item	76 (17)	71 (21)	58 (21)	55 (20)	0.73
12 hours source recall*	category	72 (21)	65 (22)	53 (27)	49 (26)	0.72
12 hours recall relative to baseline performance	item	88 (10)	81 (14)	73 (16)	65 (19)	0.76
6 days source recall	item	54 (24)	50 (25)	34 (18)	28 (17)	0.76

Table S2. Memory measures for the source memory experiment, separately for awake and sleep condition. The values are means (standard deviations in brackets) per group and condition. Note that these are all original measures, without imputations. The number of missing values per cells varies between 0-3. The level of specificity indicates whether correct recall is considered at the item-level (i.e. correct recollection of the specific face or place) or category level (i.e. face or place). *indicates the measure which formed the basis for calculating the sleep gain effect in the main analysis, subtracting the sleep from the awake performance for each individual.

Explanation of variables

Baseline indicates the percentage of correct items directly after encoding (AFC-1), before the 12 hour sleep/awake period. **12 hours source recall** indicates the percentage of correct associations after the 12 hour sleep/awake period. **The 12 hours recall relative to baseline performance** indicates correct associations at baseline and 12 hours (AFC-1-hit & AFC-2-hit) as a percentage of the baseline (AFC-1) performance, and indicates the percentage of remembered items from initially learned items. **6 days source recall** indicates the percentage of correct associations that were correct at all three test times (AFC1-hit & AFC-2-hit & AFC-3-hit): baseline, 12-hours, and 6 days.

Statistical tests

The values between sleep and wakefulness correlated highly, with correlations ranging from r = 0.72 to 0.76.

For each of the variables, we ran linear regression models, predicting memory from the age group (young vs old), condition (sleep vs awake), and interaction.

The subtractive sleep gain effect presented in the main text was not adjusted for baseline performance, as the exact category-level retrieval test (differentiation between faces and places without visual cues) was not administered immediately after encoding. Instead, participants completed a forced choice test, selecting the specific face or place for each item (AFC-1). In this context, a mixed linear model revealed a significant effect of age group ($F = -7.98$, $p < 0.001$) but no condition ($F = -1.52$, $p = 0.12$) or interaction effects ($F = 0.24$, $p = 0.80$), indicating no significant baseline learning differences between sleep and awake conditions (Table S2). Nevertheless, we recalculated the sleep gain measure using forced choice performance relative to baseline. This analysis confirmed greater retention after sleep compared with wakefulness (young: mean = 5%, sd = 11%; old: mean = 9%, sd = 14). This adjusted measure correlated strongly with the original sleep gain measure used in the PCA ($r = 0.69$).

For the 12 hours source recall (used in the PCA in the main text), linear mixed ANOVA revealed a main effect of condition (sleep > awake, $F = -2.71$, $p = 0.024$) and age group (young > old, $F = -8.82$, $p < 0.001$), indicating a similar sleep gain over wakefulness for both age groups (non-significant interaction; $F = 0.50$, $p = 0.676$). The results remained similar, if the scores were corrected for baseline learning performance, as well as for the 6 days recall.

Supplementary materials 6 (Sup6): Sleep architecture

Methods

Main indicators of sleep architecture were calculated per age group: sleep period time (from sleep onset to awakening), total sleep time, wake after sleep onset, and the percentages of time spent in each sleep stage (N1, N2, N3, REM). In addition, the average count of spindles per person was calculated, as well as overall spindle densities, normalized to the summed duration of NREM sleep stages N2 and N3. These values were compared between young and old age groups using independent t-tests. One participant was discarded from the global sleep architecture analysis due to bad signal after 4.5 hours or sleep.

Results

Variable	Old	Young	t (df)	Cohen's d	p-value
Sleep period time (h)	5.70 (0.73)	6.16 (0.56)	-2.99 (58)	-0.720	0.004*
Total sleep time (h)	5.10 (0.74)	5.70 (0.48)	-4.07 (52)	-0.99	<0.001*
Wake after sleep onset (min)	36 (25)	27 (20)	1.64 (62)	0.390	0.107
N1 proportion (%)	8.4 (4.1)	5.2 (2.7)	3.78 (53)	0.920	0.000*
N2 proportion (%)	55 (10)	48 (5)	3.48 (47)	0.870	0.001*
N3 proportion (%)	17 (9)	25 (6)	-4.07 (52)	-0.99	0.000*
REM proportion (%)	19 (7)	21 (5)	-1.52 (52)	-0.370	0.134
Spindle count (#)	1,223 (511)	1,340 (440)	-1.06 (65)	-0.250	0.293
Spindle density (# per minute of NREM sleep)	5.58 (1.89)	5.33 (1.65)	0.59 (64)	0.140	0.558

Table S3. Sleep architecture. Sleep variables and statistical comparisons between young (n=43) and old (n=34) age groups. The values for young and old are mean values, with standard deviations in the brackets. *marks significant difference between young and old at an alpha level of 0.05.

Supplementary materials 7: Spindle attributes per age group

Methods

To fully characterize the spindle profiles before clustering and for comparability with previous literature, mean value per attribute and participant was computed. For categorical attributes, we calculated the percentage of spindles (1) belonging to N2 sleep, (2) co-occurring with SOs (regardless of up- or down-phase), and (3) bilateral topography. All features were compared between older and younger age-groups using independent t-tests and fdr-corrected p-values across all attributes.

Results

The summary values for all features per age group are presented in Table S4, together with details on statistical tests. The differences between old and young groups in spindle attributes can be summarized as follows: older group had lower absolute power and dominant frequencies. The duration, relative power, and symmetry were similar across age groups. Older adults had lower proportion of bilateral spindles, compared with younger age group.

Regarding the temporal context, older participants' spindles tended to occur earlier in the night, likely due to younger participants sleeping longer and having more opportunities for spindle activity in the morning hours. There were no statistical differences regarding the proportional distribution of spindles with respect to sleep stage or their co-occurrence with SOs, nor between the inter-spindle interval or onset time relative to the start of the cycle.

Regarding the post-spindle signal characteristics, the older age group had lower delta power, lower alpha power, smaller 1/f exponent, and higher Katz fractal dimension. Altogether, these findings reflect higher proportion of slow frequency activity in younger people, together with reduced post-spindle signal complexity.

Variable	Old	young	t (df)	Cohen's d	p
**Spindle attributes**					
Duration [log(ms)]	-0.15 (0.04)	-0.14 (0.05)	-1.11 (75)	-0.250	0.303
absolute power (log_{10} μV^2)	1.98 (0.24)	2.17 (0.18)	-3.89 (58)	-0.930	<0.001*
relative power [log(%)]	-1.35 (0.19)	-1.30 (0.16)	-1.15 (66)	-0.270	0.303
Frequency (Hz)	11.13 (0.60)	11.70 (0.72)	-3.83 (75)	-0.860	<0.001*
Symmetry (a.u.)	0.497 (0.008)	0.493 (0.009)	2.21 (73)	0.500	0.054
**Temporal context**					
N2 proportion (%)	0.88 (0.09)	0.85 (0.10)	1.65 (75)	0.370	0.131
onset time (min)	147 (27)	188 (29)	-6.37 (73)	-1.45	<0.001*
onset in cycle [log(s)]	7.52 (0.35)	7.50 (0.38)	0.16 (73)	0.040	0.871
ISI [log(s)]	2.25 (0.29)	2.19 (0.26)	0.98 (67)	0.230	0.352
SO-concurrent (%)	0.40 (0.10)	0.35 (0.09)	2.07 (66)	0.480	0.063
**Topography**					
bilateral (%)	0.22 (0.08)	0.29 (0.09)	-3.42 (72)	-0.780	0.003*
**Post-spindle signal**					
delta power (%)	0.59 (0.06)	0.64 (0.07)	-3.35 (74)	-0.760	0.003*
theta power (%)	-2.11 (0.22)	-2.23 (0.26)	2.17 (75)	0.490	0.054
alpha power (%)	-2.73 (0.32)	-2.98 (0.37)	3.13 (74)	0.700	0.007*
sigma power (%)	-3.74 (0.30)	-3.85 (0.26)	1.74 (65)	0.410	0.119
1/f exponent	1.96 (0.20)	2.13 (0.14)	-4.11 (57)	-0.98	<0.001*
katz fractal dimension	1.92 (0.07)	1.88 (0.05)	3.26 (63)	0.770	0.005*
permutation entropy	0.719 (0.022)	0.709 (0.019)	2.19 (66)	0.510	0.054

Table S4. Spindle attributes. Average values (and standard deviations) for spindle attributes per age group, together with details about statistical testing. Young n = 43, old n = 34. *marks a fdr-corrected significant difference at an alpha value of 0.05. ISI = inter-spindle-interval, SO = slow oscillation.

Supplementary materials 8: Alternative clustering pipelines

Group normalization. For comparable distances between variables in the clustering algorithm, all variables must be normalized between zero and one. In our original analysis, the variables were normalized within each person. The advantage of this is that the final cluster centroids are less dependent on the original sample. That is, each new individual stands as an independent contribution, so that the final solution can be more generalizable across different samples. However, the limitation is that inter-individual variability is reduced, which may obscure the memory predictions. We therefore repeated the analysis after normalizing continuous variables at the group level.

With this normalization scheme, the variable significances were similar with the original solution, with stage and relative power having the strongest influence and k = 4 providing the best fit. In addition, the cluster profiles were almost identical, with the exception that the N2-bilateral cluster also included some spindles that co-occurred with the SOs, whereas the N2-SO^+ cluster consisted of only unilateral spindles. The memory results were similar to the original solution, in which the age group and proportion interaction was significant for the N2-SO^--bi cluster and the power correlated negatively with the sleep gain effect in each cluster.

Inclusion of post-spindle signal features. Initially, a set of features from the post-spindle signal of two seconds were extracted, based on the reasoning that spindles may serve as a gating events for following cortical consolidation processes. These features were later discarded, due to the perceived bias towards sleep stage classification. When including these features, sleep stage was still the variable with the largest significance, followed by other features capturing the power spectrum of the post-spindle signal (delta power, 1/f exponent, sigma power, alpha power, Katz fractal dimension, and theta power). Here, both k = 2 and k = 3 provided better fits than higher k's. With k = 3, clusters separated into stage N3 spindles, N2-SO-concurrent spindles, and a single cluster including all N2 spindles that did not co-occur with SOs. No age-

related memory associations (PC1) were significant with this solution. The sleep gain associations reflected the original results, with negative associations between spindle power and the sleep gain effect in each cluster.

Supplementary materials 9: SO-sigma coupling

Methods

The spindle-SO coupling was first calculated for all SOs using the YASA toolbox[14]. Shortly, the broadband data was first filtered in the SO-band (0.3-1.5 hz) and the instantaneous phase angle was extracted after the Hilbert transform of the -1/+1 second segments around the negative peaks. Then, the same data was filtered in the broad sigma frequency band (9-16 Hz) and for each SO, the phase angle at the time of the maximum sigma amplitude was extracted. Next, for each spindle that had previously determined to concur with the SO, we retained the SO with a negative peak closest to the spindle peak. The mean phase angle was calculated per participant and cluster using the *circular*[17] package in R and is expressed in radians with -pi/pi corresponding to the SO negative peak, 0 to positive peak, and positive and negative values indicating down- and up-phase, respectively.

For statistical testing of the distribution of the SO phase angles, the full SO cycle in radians was divided into 12 bins, and for each participant and cluster, the mean number of spindles per bin was calculated. For statistical testing, we shuffled the temporal order of the bins for each participant 1000 times, averaged over the permutation iteration for each bin, tested each bin against this permuted surrogate value using dependent t-tests, and applied false discovery rate p-value correction over the 12 bins[18]. This procedure was done separately for the old and young age groups. Further, the age differences in each cluster were tested by comparing the mean phase angles with Watson-Williams tests, which is a t-test alternative for circular data, using the *circular*[17] R package. Additionally, circular correlation between the phase angles in each of the two clusters was calculated using the *CircStats*[19] package in R. Lastly, circular-linear regression coefficients were calculated for the associations between the mean phase angle and age-related memory performance, as well as between the mean phase angle and sleep-related memory gain, using the *directional*[20] R-package.

Results

In line with previous literature, the N2-SO+ spindles preferably occurred just before and at the positive peak in young adults, with lower accounts on the down-phase (Figure S1A). The N3 spindles in the younger age group had a more uniform distribution on the up-phase, but still had lower occurrences on the down-phase than chance level. The older adults had more varied distributions, with spindles still occurring rather on the up phase, but closer to the negative peak. These relationships were also reflected by the mean phase angles across age groups (Figure S1B). Whereas these occurred at the up-phase for both clusters and age groups, the spindles in younger adults were closer to the positive peak than in the older adults both in the N2-SO+ cluster (mean phase angle old: -2.58 rad, young: -1.21 rad; Watson-Williams circular test F = 25.55, df = 1, 75, p<0.001) and the N3 cluster (old: -2.76 rad, young: -1.55, F = 10.49, df = 1, 75, p = 0.002). Note that the mean phase angles correlated between the N2-SO^+ and N3 clusters (circular r = 0.53, p < 0.001), and this relationship was preserved in both age groups when tested separately.

We tested the relationship between the SO phase angle and the memory performance using circular-linear regressions (Figure S1C). We found significant relationship between the phase angle of the N2-SO^+ cluster and the age-related memory performance captured by PC1 (R^2 = 0.07, p = 0.006), with participants with the phase angles closest to the positive peak on the up-phase showing better memory performance (panel C; upper left). However, this relationship was likely driven by the age differences in both variables, as it was not significant if tested separately in the age groups (both R^2's < 0.01, p's >= 0.827). There were no other significant memory associations neither with the PC1 nor with the sleep gain (all R's <= 0.03, p's >= 0.111). Note, however, that when tested separately in each age group, there was a relationship between the phase angle of the SOs coupled with the N3 spindles and the PC1 in the older adults (R^2 = 0.11 , p = 0.032), in

which older adults with SO phase angles closest to the negative peak had better memory performance (panel C, upper right).

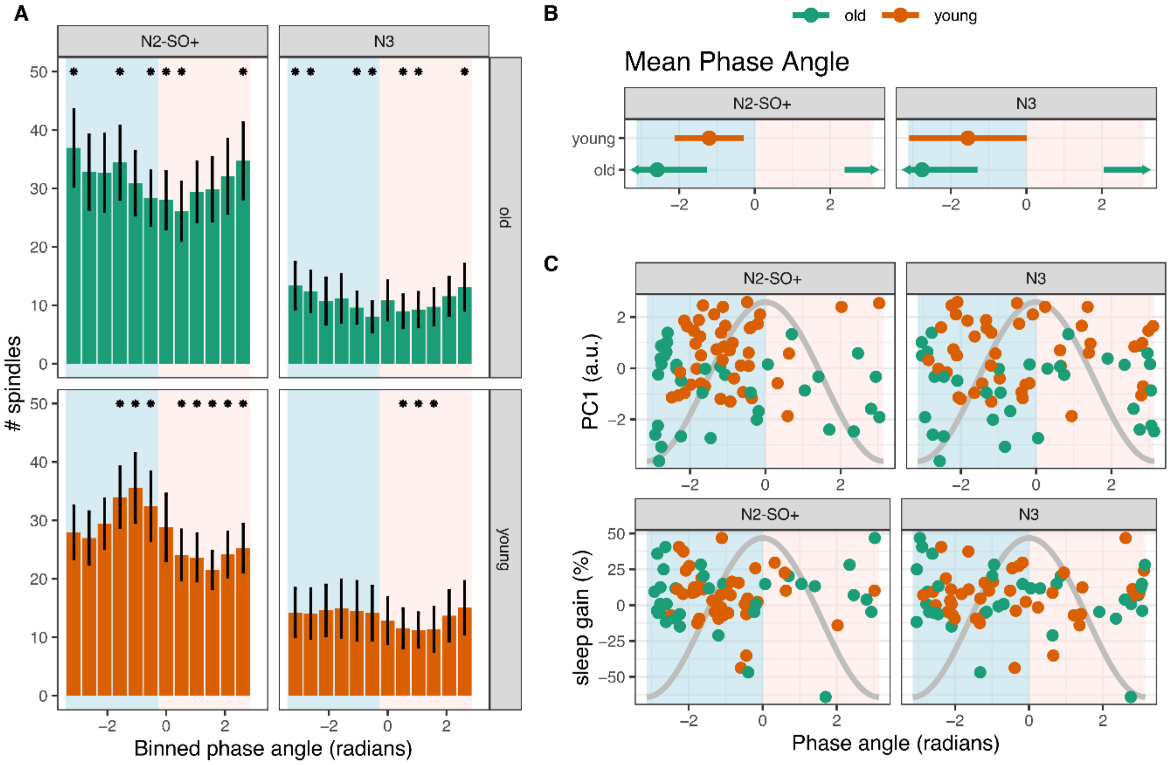

Figure S1.Spindle and slow oscillation coupling. (A) Mean number of spindles per SO phase angle bin, divided into 12 bins. The error bars indicate 95% confidence intervals. *marks significant difference when tested against within-participant permuted surrogate data. (B) Mean phase angles, with the error bars representing standard deviations. The arrows on the error bars in the older group indicate continuation over the negative peak at +/-pi. (C) Relationship between memory associations and the SO phase angle. In all subfigures, blue background shading indicates the SO up-phase and pink shading indicates the down phase.

Supplementary materials 10: Detailed statistics on age differences in spindle attributes (duration, frequency, power) in each cluster

Frequency. There were considerable frequency differences between the clusters ($F(1.73, 126.23) = 83.49$, $\eta_p^2 = 0.53$, $p < 0.001$), as well as age differences ($F(1, 73) = 13.2$, $\eta_p^2 = 0.15$, $p < 0.001$). Whereas there was also a significant interaction of age and cluster interaction ($F(1.73, 126.23) = 5.75$, $\eta_p^2 = 0.07$, $p = 0.006$), all pairwise comparisons still indicated higher frequencies in younger than older in each cluster (all d's >= |0.93|, all p's <= 0.016). Similarly, differences in frequencies between clusters in both age groups were all significant (young: all d's >= 5.64, all p's <= 0.001; old: all d's >= |2.15|, all p's <= 0.039). Specifically, older adults had the biggest frequency difference in the N2-SO^--bi cluster, compared with the young age group.

Power. There were considerable cluster ($F(1.79, 230.9) = 108.93$, $\eta_p^2 = 0.60$, $p < 0.001$), age ($F(1, 73) = 21.80$, $\eta_p^2 = 0.23$, $p < 0.001$), and sex ($F(1, 73) = 16.69$, $\eta_p^2 = 0.18$, $p < 0.001$; women > men) differences in absolute spindle power, superseded by significant cluster and age interaction ($F(1.79, 230.9) = 4.47$, $\eta_p^2 = 0.06$, $p = 0.016$). Despite this significant interaction, the power was higher in the younger age group than older in all clusters (all d's >= |0.74|, all p's <= 0.002). Generally, the power was the highest in the N2-SO^--bi cluster, followed by the N2-SO^+, N3, and finally the N2-SO^--uni cluster. All pairwise comparisons were significant in both age groups (young: all d's >= |0.16|, all p's < 0.001; old: all d's >= |0.13|, all p's <= 0.009), except for between N2-SO^+ and N3 cluster in the older adults (d = 0.07, p = 0.008).

In sum, power was larger in young than old age group in all clusters. However, power differences between clusters were generally stronger for the young than for the older age group.

Duration. The spindle durations were longest in all the stage N2 clusters ('N2-SO^--bi > N2-SO^+ > N2-SO^--uni > N3; main effect: $F(2.27, 165.72) = 355.88$, $\eta_p^2 = 0.83$, $p < 0.001$; pairwise comparisons: all d's >= |0.73|, all p's < 0.001). There were no age ($F(1, 73) = 0.15$, $\eta_p^2 = 0.002$, $p = 0.705$) or sex differences ($F(1, 73) = 3.35$, $\eta_p^2 = 0.04$, $p = 0.071$).

Supplementary materials 11: Spindle power and sleep gain associations by cluster

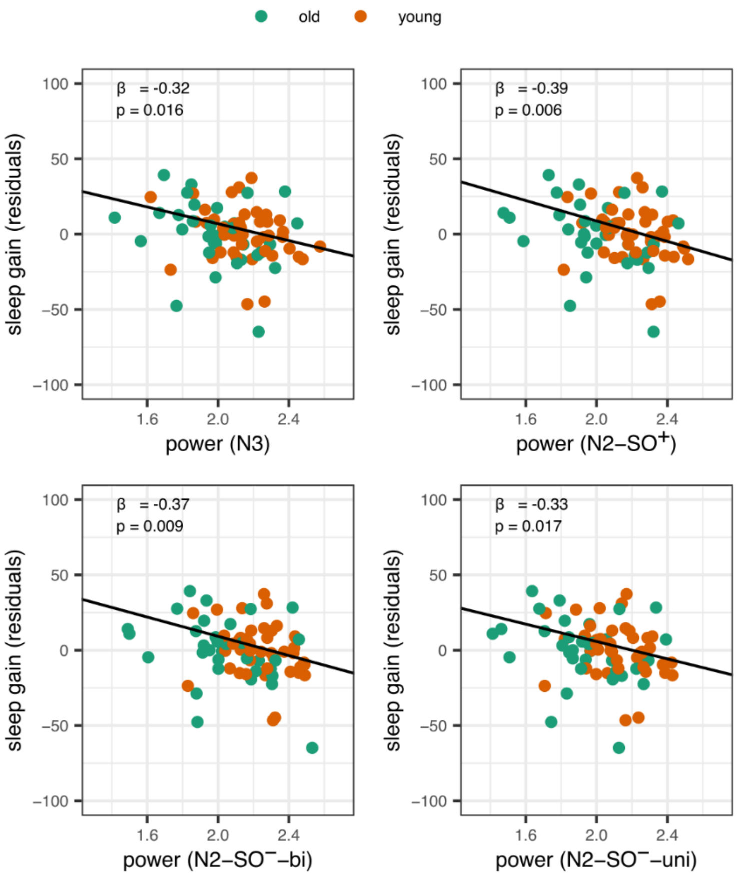

Figure S2. Sleep gain and spindle power by cluster. The sleep gain memory scores are residualized for sex and age group, but the regression lines and text annotations represent the intercept, slope, beta, and p-values from the original models. The power is given in $log_{10}(\mu V^2)$.

References

1. Johnson MK, Hashtroudi S, Lindsay DS. Source monitoring. *Psychol Bull*. 1993;114(1):3-28. doi:10.1037/0033-2909.114.1.3

2. Ness HT, Folvik L, Sneve MH, et al. Recalled through this day but forgotten next week?—retrieval activity predicts durability of partly consolidated memories. *Cerebral Cortex*. 2024;34(6):bhae233. doi:10.1093/cercor/bhae233

3. Ness HT, Folvik L, Sneve MH, et al. Reduced Hippocampal-Striatal Interactions during Formation of Durable Episodic Memories in Aging. *Cerebral Cortex*. 2022;32(11):2358-2372. doi:10.1093/cercor/bhab331

4. Stickgold R, Walker MP. Sleep-dependent memory triage: evolving generalization through selective processing. *Nat Neurosci*. 2013;16(2):139-145. doi:10.1038/nn.3303

5. Paller KA, Creery JD, Schechtman E. Memory and Sleep: How Sleep Cognition Can Change the Waking Mind for the Better. *Annual Review of Psychology*. 2021;72(Volume 72, 2021):123-150. doi:10.1146/annurev-psych-010419-050815

6. Zhao X, Chen PH, Chen J, Sun H. Manipulated overlapping reactivation of multiple memories promotes explicit gist abstraction. *Neurobiol Learn Mem*. 2024;213:107953. doi:10.1016/j.nlm.2024.107953

7. Sunderland A, Harris JE, Baddeley AD. Do laboratory tests predict everyday memory? A neuropsychological study. *Journal of Verbal Learning and Verbal Behavior*. 1983;22(3):341-357. doi:10.1016/S0022-5371(83)90229-3

8. Sunderland A, Harris JE, Gleave J. Memory failures in everyday life following severe head injury. *Journal of Clinical Neuropsychology*. 1984;6(2):127-142. doi:10.1080/01688638408401204

9. Donoghue T, Haller M, Peterson EJ, et al. Parameterizing neural power spectra into periodic and aperiodic components. *Nature neuroscience*. 2020;23(12):1655-1665.

10. Vallat R. raphaelvallat/antropy. Published online February 4, 2025. Accessed February 7, 2025. https://github.com/raphaelvallat/antropy

11. Lau ZJ, Pham T, Chen SHA, Makowski D. Brain entropy, fractal dimensions and predictability: A review of complexity measures for EEG in healthy and neuropsychiatric populations. *Eur J Neurosci*. 2022;56(7):5047-5069. doi:10.1111/ejn.15800

12. Helfrich RF, Lendner JD, Knight RT. Aperiodic sleep networks promote memory consolidation. *Trends Cogn Sci*. 2021;25(8):648-659. doi:10.1016/j.tics.2021.04.009

13. Ma Y, Shi W, Peng CK, Yang AC. Nonlinear dynamical analysis of sleep electroencephalography using fractal and entropy approaches. *Sleep Med Rev*. 2018;37:85-93. doi:10.1016/j.smrv.2017.01.003

14. Vallat R, Walker MP. An open-source, high-performance tool for automated sleep staging. Peyrache A, Büchel C, Bagur S, eds. *eLife*. 2021;10:e70092. doi:10.7554/eLife.70092

15. Goh C, Hamadicharef B, Henderson GT, Ifeachor EC. Comparison of Fractal Dimension Algorithms for the Computation of EEG Biomarkers for Dementia. In: 2005. Accessed February 7, 2025. https://inria.hal.science/inria-00442374

16. Esteller R, Vachtsevanos G, Echauz J, Litt B. A comparison of waveform fractal dimension algorithms. *IEEE Transactions on Circuits and Systems I: Fundamental Theory and Applications*. 2001;48(2):177-183. doi:10.1109/81.904882

17. Lund U, Agostinelli C, Arai H, et al. circular: Circular Statistics. Published online August 29, 2024. Accessed April 22, 2025. https://cran.r-project.org/web/packages/circular/index.html

18. Muehlroth BE, Sander MC, Fandakova Y, et al. Precise Slow Oscillation-Spindle Coupling Promotes Memory Consolidation in Younger and Older Adults. *Sci Rep*. 2019;9(1):1940. doi:10.1038/s41598-018-36557-z

19. Lund S plus original by U, Agostinelli R port by C. CircStats: Circular Statistics, from “Topics in Circular Statistics” (2001). Published online July 1, 2018. Accessed April 22, 2025. https://cran.r-project.org/web/packages/CircStats/index.html

20. Tsagris M, Athineou G, Adam C, et al. Directional: A Collection of Functions for Directional Data Analysis. Published online February 11, 2025. Accessed April 22, 2025. https://cran.r-project.org/web/packages/Directional/index.html
